# Mirroring spinal cord lesion secondary to B12 deficiency

**DOI:** 10.11604/pamj.2017.26.130.11853

**Published:** 2017-03-07

**Authors:** Youssouf Benmoh, Ahmed Bourazza

**Affiliations:** 1 Department of Neurology, Military Hospital MOHAMED V, Rabat, Morocco

**Keywords:** B12 deficiency, combined sclerosis, spinal cord, MRI

## Image in medicine

A 26 year-old girl, previously healthy, admitted for progressive weakness of both lower limbs, with permanent paresthaesia evolving for 6 months. Clinical examination found spastic paraparesis with proprioceptive ataxia. The MRI showed bilateral and symmetrical lesions in the cervical spinal cord. Lesions were T1-isointense and T2 hyperintense, located on both lateral and posterior funiculus without enhancement. Investigation revealed deep vitamin B12 deficiency (27pg/ml), macrocytosis, with positive parietal cell-antibody and fundic gastritis. Patient was treated with intra muscular hydorxycobalamin. Evolution was favorable with regression of weakness and parethaesia of both lower limbs, and persistence of discreet ataxia. Cobalamin deficiency is common in the elderly. It causes hematologic, digestive and neurological disorders. Neurological signs are dominated by combined sclerosis of the spinal cord, and peripheral neuropathy. MRI is useful in front of myelopathy, showing an enlargement or T2-hyperintense lesion of the posterior funiculus.

**Figure 1:**
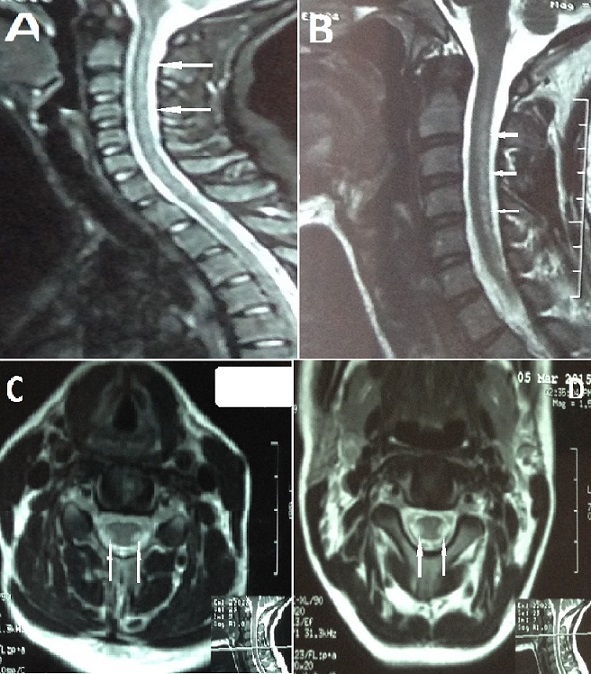
(A,B) MRI of the spinal cord, in sagittal T2 weighted image showing an increased signal intensity extended to the whole cervical spinal cord; (C,D) MRI of the spinal cord, in axial T2 weighted image, showing increased bilateral and symmetrical signal intensity of lateral and posterior funiculus.

